# Mechanical Properties of Structural Components in Hastelloy X Joints Brazed with Ni-Pd-Cr-B-Si Alloy

**DOI:** 10.3390/ma16031115

**Published:** 2023-01-28

**Authors:** Michał Baranowski, Jacek Senkara

**Affiliations:** Faculty of Mechanical and Industrial Engineering, Warsaw University of Technology, Narbutta 85, 02-524 Warsaw, Poland

**Keywords:** brazing, Hastelloy X, Ni-Pd-Cr-B-Si, structural components, instrumented indentation test, mechanical properties

## Abstract

The brazing of structural high-temperature-resistant nickel alloys is a predominant method in manufacturing jet engines in the aircraft industry. Ni-Cr-base brazing filler metals (BFMs) containing B and Si as the melting point depressants are used for this purpose. The presence of the latter can lead to the formation of brittle constituents in the joints, decreasing their strength, toughness and creep resistance. The structures of Hastelloy X nickel superalloy joints brazed with Palnicro 36M BFM are presented in this paper along with the mechanical properties of their particular phases as a function of brazing time. Indentation hardness, Martens hardness, reduced modulus and creep coefficient were measured using the instrumented indentation method. The elastic part of the indentation work was also calculated. Pd forms an unlimited solution with Ni, but its high content in BFM does not fundamentally change the general joint structure known from other Ni-superalloy–Ni-BFM systems. However, new Pd-containing phases are emerging. The hardest components were Ni-B and Cr-B boride phases and Pd-Ni-Si phase in MZ and the boundary of DAZ and BM. MZ reduces the plasticity of a joint to the highest extent. The hardness of particular parts in the joints and the elastic portion of the indentation work decreased with the increase in brazing time, while the reduced modulus of the indentation contact and indentation creep increased. The results of indentation creep measurements indicate that all structural components of the joints were less susceptible to creep than the parent material at room temperature.

## 1. Introduction

Brazing is one of the special processes that significantly affect the quality of a final product. One of the important areas of application of this process, apart from the automotive and energy sectors, is the aviation industry. The family of Ni-Cr alloys (BNi group) is used as brazing filler metals (BFM) for joining aircraft engine components made of Ni superalloys. Usually, such BFMs contain some amount of temperature depressants—boron and silicon—that provide a lower melting range for these alloys. B and Si further promote the reduction of oxides and also enable the production of amorphous brazing foils from the alloy. Boron reduces the surface tension of liquid Ni-Cr BFMs; it is also an element of high diffusivity with a tendency to segregate at grain boundaries. Boron also increases the creep resistance of joints, whereas silicon improves resistance to oxidation and sulfation. However, they cause the formation of brittle phases in the joints as well, which have a detrimental effect on their mechanical properties (see overviews [1,2], for instance). An analysis of the relationship between the structure of joints brazed with Ni-Cr BFMs and their properties is presented in papers [3,4,5,6].

The mechanism of joint formation is complex and the duration of its particular stages may vary depending on the time of the process. It has been the subject of a number of studies, especially for the most commonly used BNi2 and BNi3 [7,8,9,10,11,12], and other BFMs based on Cr-Ni [13,14,15]. There is an agreement that, in general, the BFMs in question create the joint structures in which three main zones can be distinguished. As a result of intensive diffusion of B from BFM to the base material (BM) a diffusion-affected zone (DAZ) is formed. Next, the solid solution phase (SSP) zone, also called the isothermal solidification zone, adjoins the DAZ. Its formation is explained by the isothermal solidification phenomenon due to the interdiffusion between BM and BFM that reduces the B and Si content in the liquid. During cooling, along the central region of the joint, a multiphase zone (MZ), or athermal solidification zone, arises. The eutectic nature of this zone with brittle phases is pointed out. It is believed that the presence of MZ is related to the formation of the primary solidification and the segregation of elements. Appropriate selection of brazing parameters allows obtaining a joint without or with a limited amount of brittle phases along the central region. A number of research works have been devoted to this aspect of brazing with the use of Ni-Cr-base BFMs. The existence and amount of undesirable phases can be limited by the suitable choice of the joint clearance, the proper selection of brazing parameters (i.e., temperature and time [16,17,18,19]) to prolong isothermal solidification stage and/or the followed post-heat treatment [20,21]. A method for optimizing the brazing temperature allowing the shortest isothermal solidification time to be obtained, considering a widening of the gap due to the substrate dissolution during brazing, was proposed recently by Corbin and Tadgell [22]. It was applied to the investigation of the Inconel 718–Ni-Pd-Cr-B-Si BFM pair [23].

It is also worth noting that the formation of the joint, and consequently its structure and properties, are influenced not only by the type of BFM and applied process parameters, but also by the character of the brazed material. This is related to both the condition of its surface state [24,25] and also the type of interaction with liquid BFM: dissolution of the substrate and kinetics of diffusion of components into it [26,27].

Along with BNi filler metals, Ni-Cr-B-Si alloys with the addition of palladium up to 46 wt.% were introduced, and are currently commercially produced for joining some representatives of the Inconel and Hastelloy groups of Ni superalloys. An addition of 35 wt.% level of Pd into Ni-Cr-base BFM has been proposed first as a replacement of gold in the Au-18 wt.% BFM of the BAu family [28]. Aside from reducing the amount of precious metals, it is believed that Pd presence improves the wettability and spreadability of liquid BFMs and has a positive effect on the ductility of joints, and their resistance to creep at elevated temperatures [28,29,30,31].

Analysis of the relevant binary and ternary phase equilibrium systems indicates that, despite of the creation of Ni-Pd solid solution in an unlimited range of concentrations [32], Pd supplement in Ni-Cr-B-Si BFMs does not protect against the formation of brittle phases. Moreover, besides typical borides/silicides, a number of binary Pd-Si, Pd-B and ternary Ni-Pd-Si intermediate phases have been reported [33,34,35,36,37,38].

Multi-component nickel superalloys of γ (FCC) structure for high-temperature applications (Inconel, Hastelloy, Haynes, Nimonic groups) can be divided into solution-strengthened materials (Inconel 617 and 625, Hastelloy X, for example) and γ’ or γ’’ precipitation phase-reinforced ones (Haynes 242, 263, 282, Inconel 718, Waspaloy). This affects both their use and high-temperature processing, including welding and brazing. Various aspects of their behaviour at elevated temperatures can be found in papers [39,40] for Inconel 617 alloy and [41] for Haynes 282 alloy. The weldability of most Ni superalloys is limited by the possibility of defects and discontinuities in welds [42,43,44]; therefore, brazing is preferred in responsible aerospace applications.

In this work, Hastelloy X brazed joints prepared with the use of Ni-Cr-base BFM with 36 wt.% addition of Pd (Palnicro 36M) were examined. Measurements of mechanical properties of structural components revealed in the joints were taken using the instrumented indentation test (IIT).

## 2. Materials and Methods

**Figure 1:**
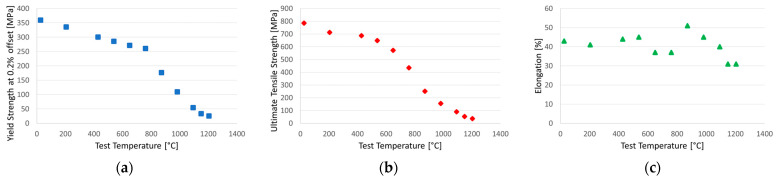
Average tensile data of Hastelloy X (2.8 mm thick sheet; heat-treated at 1177 °C and rapid cooled): yield strength at 0.2% offset (**a**), ultimate tensile strength (**b**), elongation in 50.8 mm (**c**) [45].

Solution-strengthened Hastelloy X is a Ni-base superalloy with exceptional resistance in oxidizing atmospheres (up to 1200 °C). It has very good heat resistance and creep resistance even up to 800 °C, thanks to which it is used as a constructional material in combustion chambers, afterburners and honeycomb seals in aircraft engines. It also manifests extraordinary resistance to stress corrosion cracking in petrochemical applications. It has good ductility and can be cold-worked [45,46,47,48]. Selected mechanical properties of this material as a function of temperature are presented in Figure 1.

Palnicro 36M BFM is characterized by excellent creep resistance at elevated temperatures and also found an application in the aviation industry. Its solidus–liquidus temperature range is 820–960 °C and recommended brazing temperatures are 970–1050 °C. Typical applications of this BFM include aero engine compressor vanes and stators, aerospace fuel-line assemblies, power supply surge arrestors and automotive components [29,30].

Hastelloy X Ni-superalloy was used in the form of 0.8 mm thick plates in this study. Ni-Pd-Cr-B-Si (Palnicro 36M) amorphous foil with a thickness of 0.05 mm was applied as BFM, in delivery conditions. The nominal chemical composition, solidus and liquidus temperatures of both materials are presented in Table 1.

**Figure 2:**
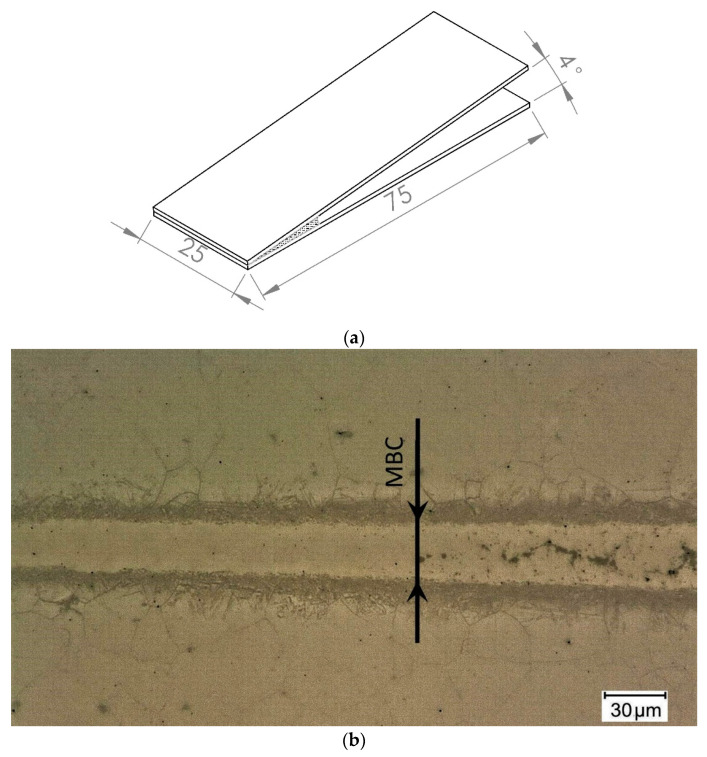
The wedge gap sample: scheme of configuration (**a**) and the cross section of the joint with maximum brazing clearance marked (**b**).

Wedge gap and flat gap specimens were made in a brazing process in a vacuum furnace < 10^−3^ mbar. Surface roughness of Hastelloy X substrates was Ra = 0.11 μm, and the coupons were cleaned in an ultrasonic scrubber in acetone prior to brazing. The assemblies were heated up in special fixtures with a 15 °C/min heating rate up to the 995 °C brazing temperature, held and cooled in the furnace. Four brazing times were applied: 10, 20, 30 and 60 min. Two samples were made for each wedge test and four in the flat joints variant, for each of the four brazing times (24 samples in total). Configuration of the first type of samples is shown in Figure 2a.

The structural and indentation tests were carried out on specimens in the plane perpendicular to the joints after the standard metallographic procedure. Microstructure observations were made using a digital optical microscope with the ability for basic dimension measurements. The maximum brazing clearance (MBC) was measured in wedge gap samples, for which inclusions of hard phases do not exist yet in the center. Figure 2b shows an example of such a sample with MBC value marked.

The imaging of the tested samples was continued using a scanning electron microscopes (SEM)—JEOL JSM-IT100 and Thermofisher scientific Axia ChemiSEM. Identification of elements was performed using the energy dispersive spectroscopy (EDS) method. An EDS-equipped field-emission scanning electron microscope (FESEM)—JEOL JSM-7600F—was additionally used for the identification of fine grain phases.

The instrumented indentation test (IIT) was used to determine selected mechanical properties of structural components in the joints. Microhardness tester with Vickers diamond indenter (α = 136° ± 0.2°) was applied. Based on the registration of indenter penetration into the material with the loading assumed, the hardness, elasticity and creep parameters were determined. According to the ISO 14577-1 standard [49], indentation hardness (H_IT_), Martens hardness (H_M_), reduced modulus of the indentation contact (E_r_), elastic part of indentation work (η_IT_) and indentation creep (C_IT_) were defined. During the first stage of the measurements, the joints were tested with a 30 mN maximum load (P_max_) of the indenter, 200 mN/min loading/unloading rate (v) and 10 s holding time. The holding time was extended to 50 s for the indentation creep test. Further measurements were taken with P_max_ and ν increasing up to 1 N and 2 N/min, respectively. The results are presented as the average of three measurements for each variant.

## 3. Results

### 3.1. Microstructure of Hastelloy X–Palnicro 36M Joint

#### 3.1.1. Wedge Test

**Figure 3:**
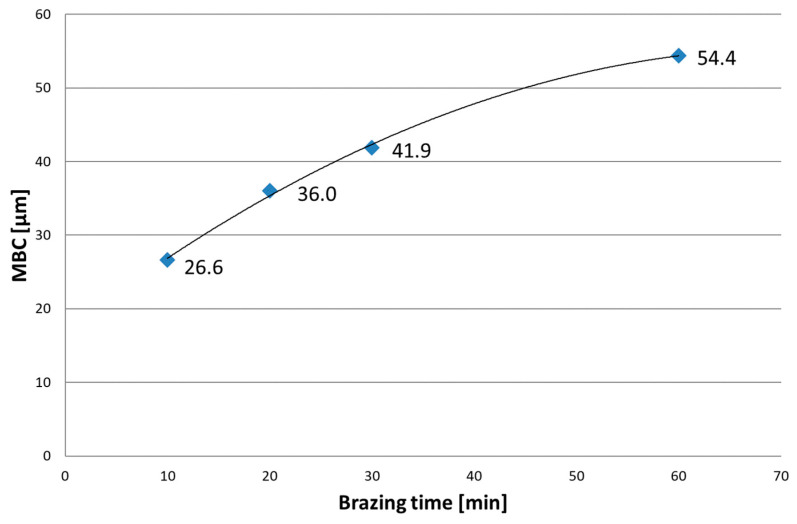
Impact of brazing time on MBC in Hastelloy X–Ni-Pd-Cr-B-Si joints.

As expected, in Hastelloy X joints brazed with Palnicro 36M, precipitations of phases are observed in the central part of the joints. Depending on the brazing parameters, the gap sizes were recorded for which the joints had a single-phase structure. Figure 3 demonstrates the results of MBC measurements as a function of brazing time.

**Figure 4:**
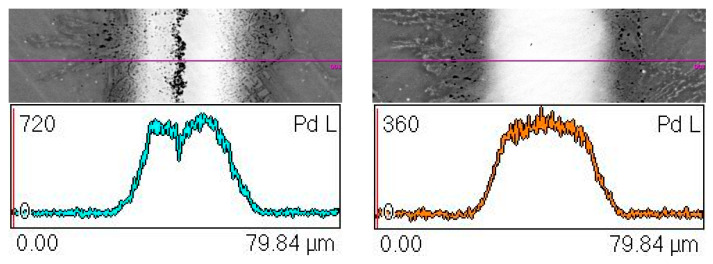

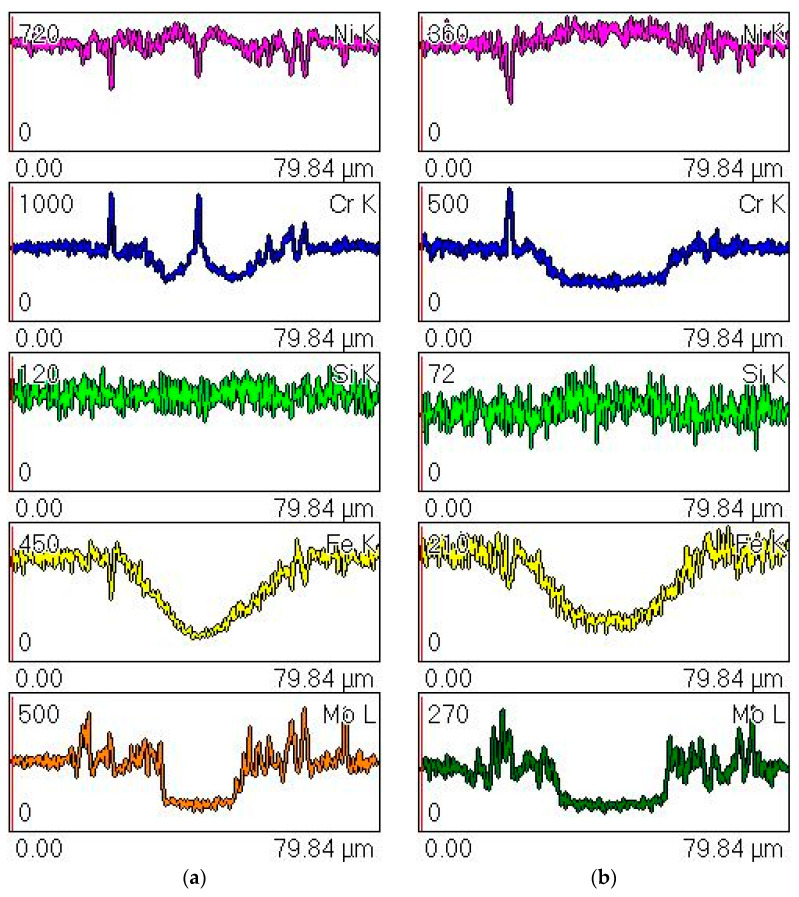
Structure of Hastelloy X–Ni-Pd-Cr-B-Si joint and linear distributions of element concentration along the marked line after 10 min (**a**) and after 30 min (**b**) brazing time at 995 °C.

The following Figure 4 shows the structures of joints with linear distributions of element concentration after 10 min and 30 min brazing time. They were obtained in cross-sections at the same distance from the beginning of the samples and have a similar width. The samples were selected because of their structure, which differs in the occurrence of MZ in the joint after 10 min of brazing time (Figure 4a), while this zone is not present in the sample brazed for 30 min (Figure 4b).

#### 3.1.2. Flat Joints

**Figure 5:**
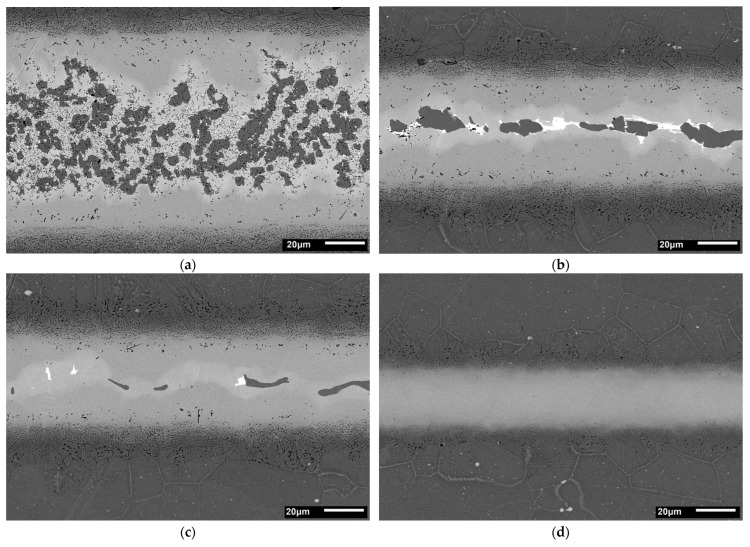
Structures of Hastelloy X–Palnicro 36M joints obtained after 30 min brazing at 995 °C for different gap sizes, approximately 90 μm (**a**), 55 μm (**b**), 45 μm (**c**) and 30 μm (**d**).

**Figure 6:**
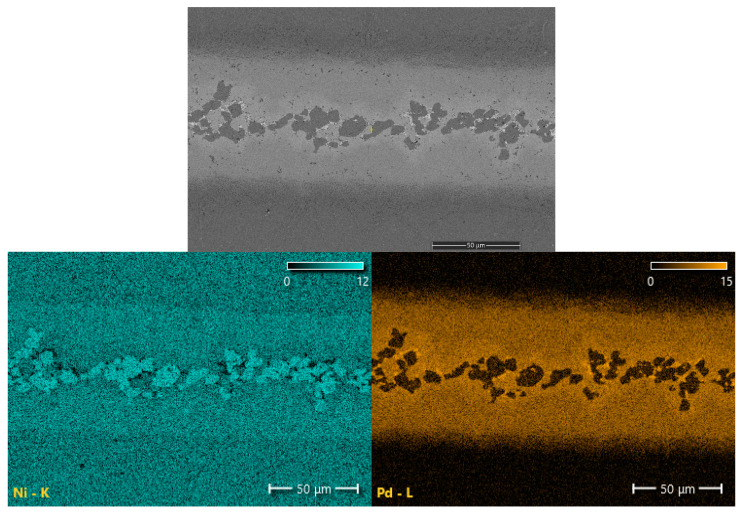

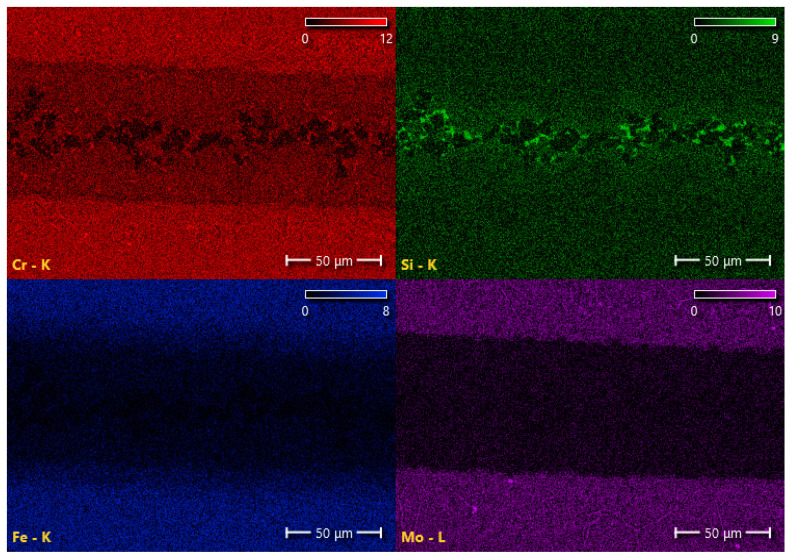
Hastelloy X–Palnicro 36M joint obtained after 30 min brazing at 995 °C: SEM micrograph and EDS maps of elements’ distribution.

**Figure 7:**
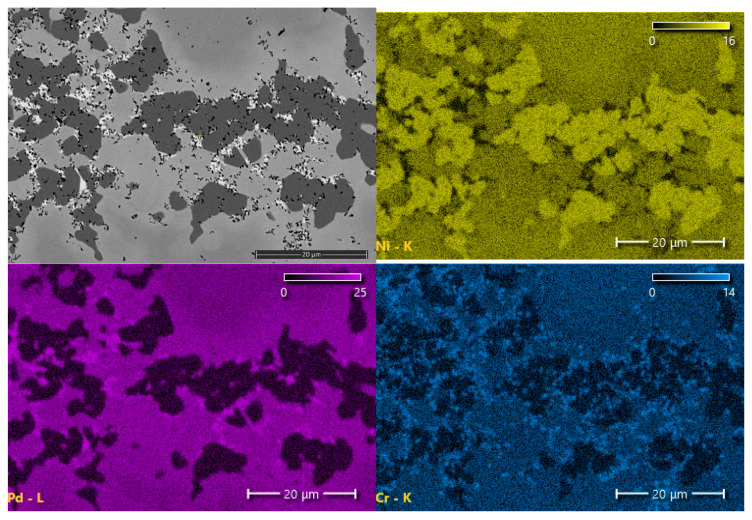

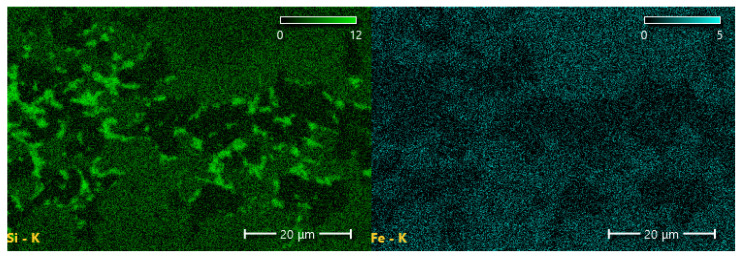
MZ in Hastelloy X–Palnicro 36M joint obtained after 30 min brazing at 995 °C: SEM micrograph and EDS elemental distribution maps.

On the basis of the wedge test, a set of samples brazed for 30 min was prepared with different gap sizes. Metallographic and SEM analysis of joints’ cross-sections confirmed the results obtained in the wedge test: the presence and morphology of MZ differ for individual BFM distances. Structures of appropriate samples are presented in Figure 5. and the maps of the distribution of elements in Figure 6. There is a clear dependence of the existence of the MZ zone and its morphology on the size of the brazing gap. In turn, surface distributions of elements in the MZ zone are shown at higher magnification in Figure 7.

**Figure 8:**
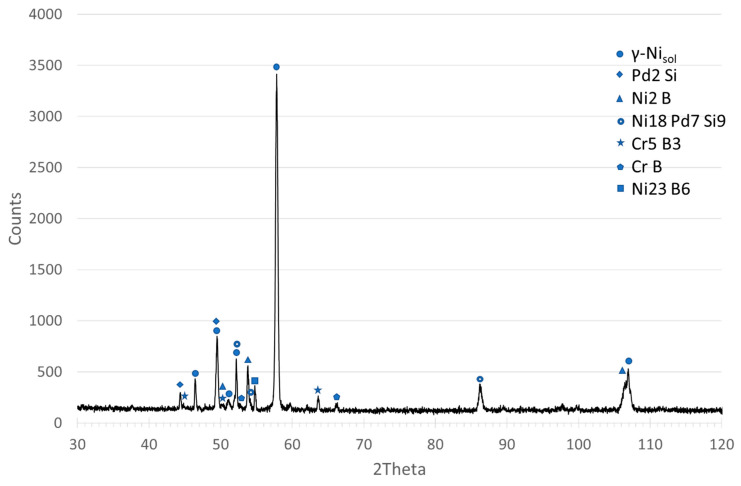
XRD patterns of MZ in Hastelloy X–Palnicro36 (Cobalt lamp WL—1.78897, counting time—5s, step—0.025 degrees).

Examinations of the chemical composition of the joint components and their mechanical properties were carried out for the sample in which three basic zones can be clearly distinguished: MZ, SSP and DAZ. The results of SEM–EDS investigations of the MZ and SSP zone are presented in Table 2. In turn, FESEM–EDS was used for the analysis of fine inclusions and for the verification of B content (the latter is not an easy task in the case of Ni superalloys [50]). In the latter, a reduced acceleration voltage of 5 kV was used, which is considered optimal for the analysis of boron and other light elements with the EDX method [51]. The results indicate the presence of boron in the dark gray phase and in the fine black phase (see MZ micrograph in Figure 7), however, due to the large EDX measurement error for this element, quantitative results of the concentration of elements in both phases could not be presented. To verify the results, an XRD phase analysis of the MZ of the joint was also performed, the results of which indicate the existence of chromium and nickel borides in this zone, and also the presence of a Ni-Pd-Si phase (Figure 8).

On the other hand, DAZ was the subject of an earlier work [52] in which combined X-ray diffraction (XRD) and SEM-EDX method was employed. The following phases were revealed there: MoNi_3_, NiFe, CrNi, Cr_5_B_3_ and Cr-Mo-B. The possibility of the occurrence of phases based on Cr_3_NiB_6_ and Mo_6_(Ni0.75Si_0_._25_) intermetallic compounds was also pointed out.

**Figure 9:**
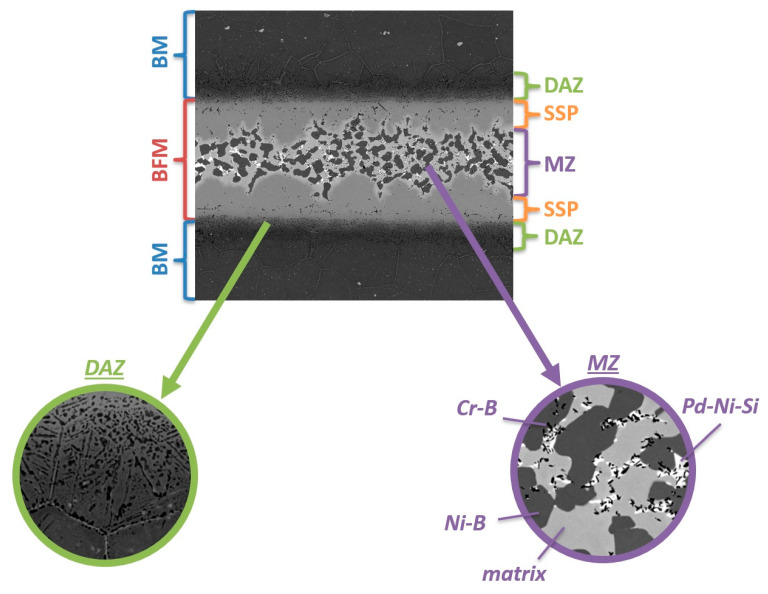
Summarized structure of Hastelloy X–Palnicro 36M brazed joint designed for mechanical testing.

Figure 9 presents a summary of the disclosed structural components of the Hastelloy X–Palnicro 36M joint that was subjected to further indentation tests. The selected sample is not the optimal structure (wide MZ with numerous precipitates of brittle phases), but was deliberately selected due to the abundance and diversity of its structural components.

### 3.2. Mechanical Properties

**Figure 10:**
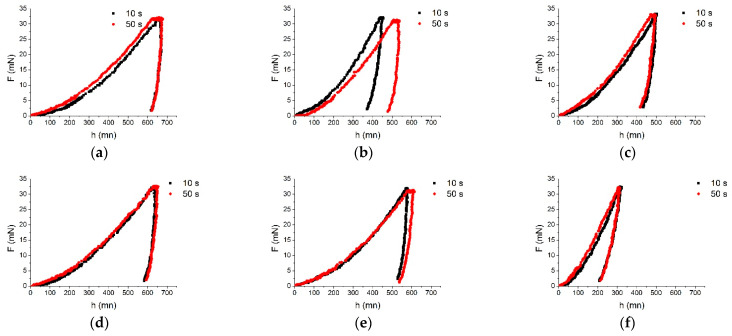

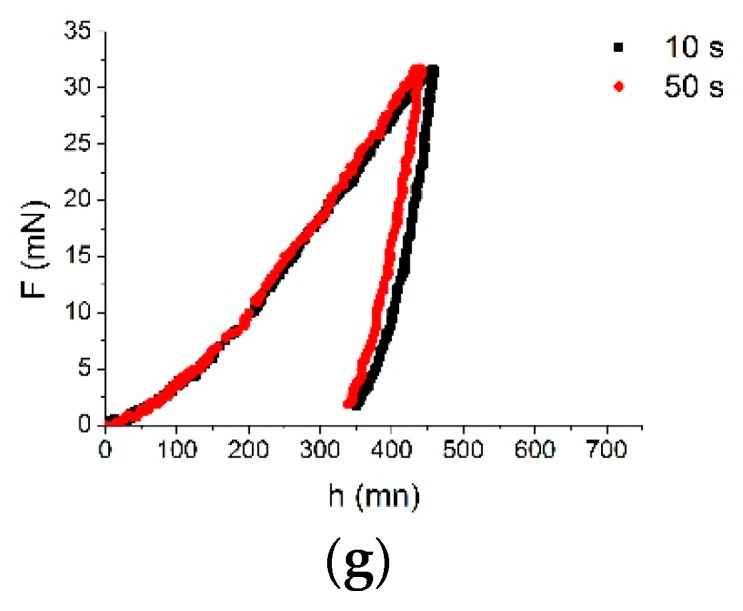
Typical load vs. displacement plots obtained for 10 s and 50 s holding time for: BM (**a**), BM-DAZ (**b**), DAZ-SSP (**c**), SSP (**d**), matrix in MZ (**e**), Ni-B (MZ) (**f**), Pd-Ni-Si (MZ) (**g**).

**Figure 11:**
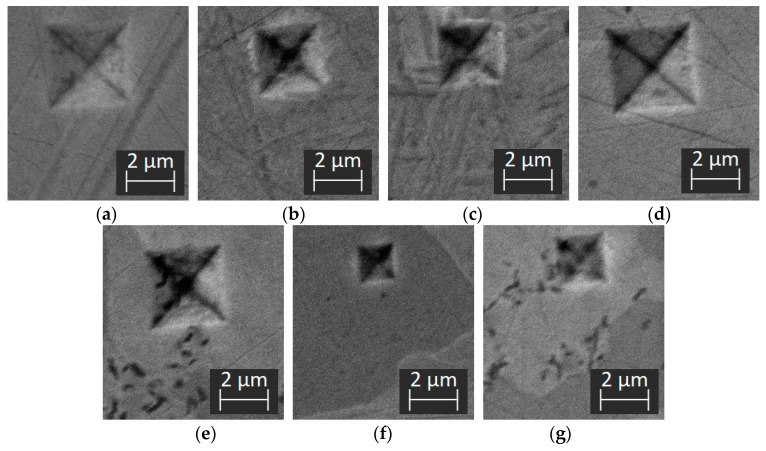
SEM micrographs of the indent marks on: BM (**a**), BM-DAZ (**b**), DAZ-SSP (**c**), SSP (**d**), matrix in MZ (**e**), Ni-B (MZ) (**f**), Pd-Ni-Si (MZ) (**g**).

Measurements were carried out for the BM, DAZ, SSP and MZ areas. All results presented are the average values of three tests. The indentation depth changed in the range 306–681 nm. The load–displacement plots for both loading times of 10s and 50s for each structure component are presented in Figure 10, while the SEM micrographs in Figure 11.

Although four phases were identified in MZ, due to the small size of Cr-B precipitations, measurements for this phase were omitted. The tests were carried out for the matrix, Ni-B, and Pd-Ni-Si phases. The SSP zone adjacent to MZ constitutes a uniform solid solution and it was a subject of tests. In turn, DAZ arises as a result of BM–BFM interaction leading to the creation of fine precipitations. Taking into account the size of the precipitations and applied 30 mN load in IIT on the other, the results for DAZ should be treated as the average values of Mo-Cr-B, another hard phase mentioned earlier and revealed in [52], and a more plastic matrix. The measurements in DAZ were performed for its two border regions marked as BM–DAZ and DAZ–SSP. 

**Figure 12:**
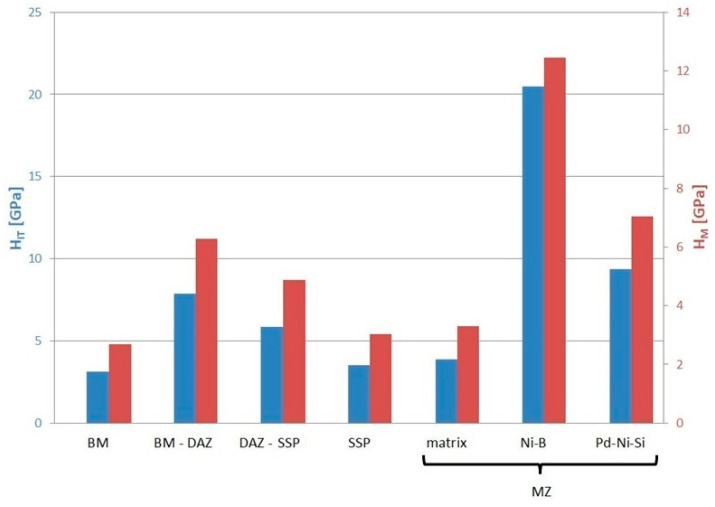
Indentation hardness and Martens hardness for structure elements of Hastelloy X–Palnicro 36M brazed joint.

Hardness results obtained (i.e., indentation hardness and Martens hardness) are shown in Figure 12.

**Figure 13:**
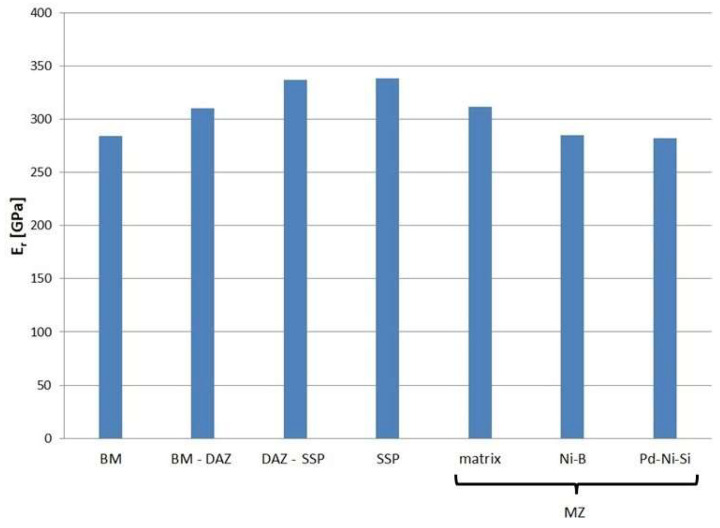
Reduced modulus of the indentation contact of structure elements in the joint.

The tests conducted also allow the withdrawal of other mechanical properties from the load–displacement P(h) curves. The measured reduced modulus of the indentation contact may be treated as the elastic behavior indicator of the microstructure elements of brazed joints. The results are presented in Figure 13.

**Figure 14:**
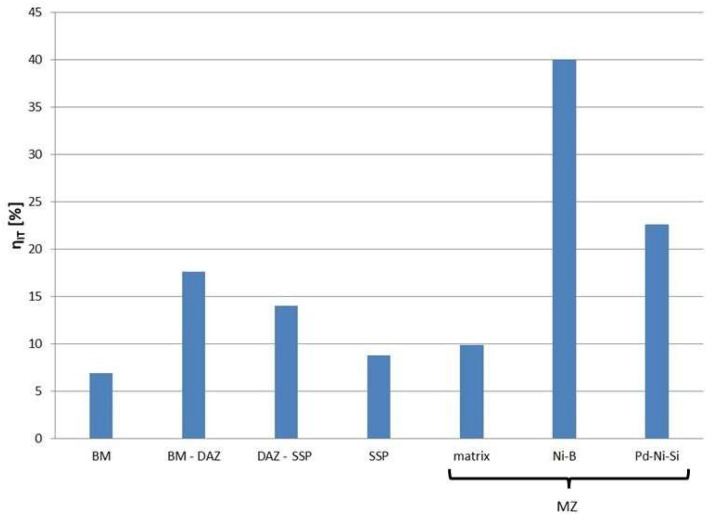
The relative percentage of elastic part of the indentation work (elastic to total work ratio) for structure elements of Hastelloy X–Palnicro 36M brazed joint.

As another elastic behavior indicator, the values of elastic work to the total work ratio η_IT_ were applied. This calculation was possible since P(h) curves characterize the elastoplastic nature of the deformation. The area under the loading curves represents the total accumulated work of material deformation, whereas the area under the unloading curves is defined as the elastic response (elastic work). Both these areas were measured from the charts and their ratio is presented in Figure 14.

**Figure 15:**
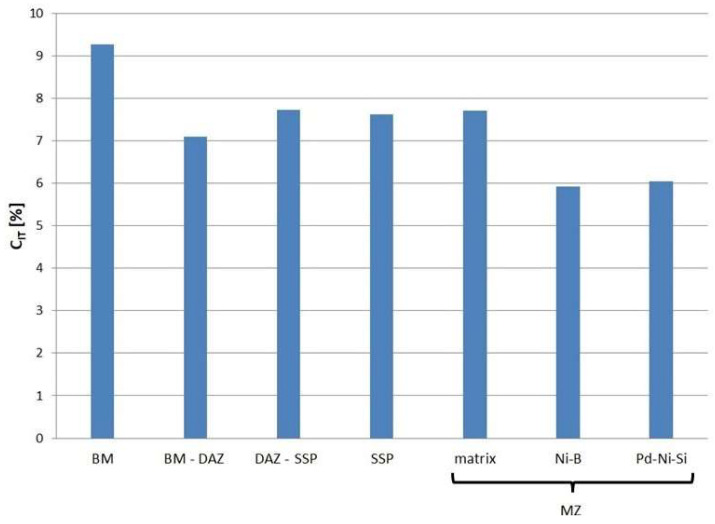
Indentation creep of structure elements in Hastelloy X–Palnicro 36M brazed joints.

The IIT method made it possible to determine the indentation creep for individual microstructure elements of the tested joint. An indicator of creep susceptibility is the C_IT_ ratio, which describes the percent change in indention depth over time under a constant load. The results of the calculations are presented in Figure 15.

**Figure 16:**
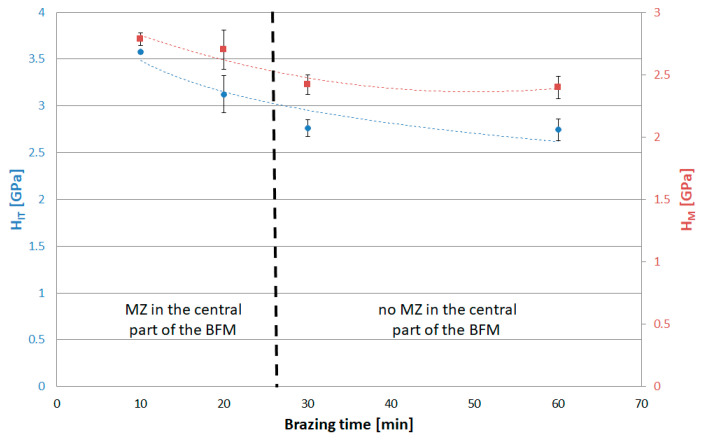
Indentation hardness and Martens hardness for Hastelloy X–Palnicro 36M joints by 1 N load.

**Figure 17:**
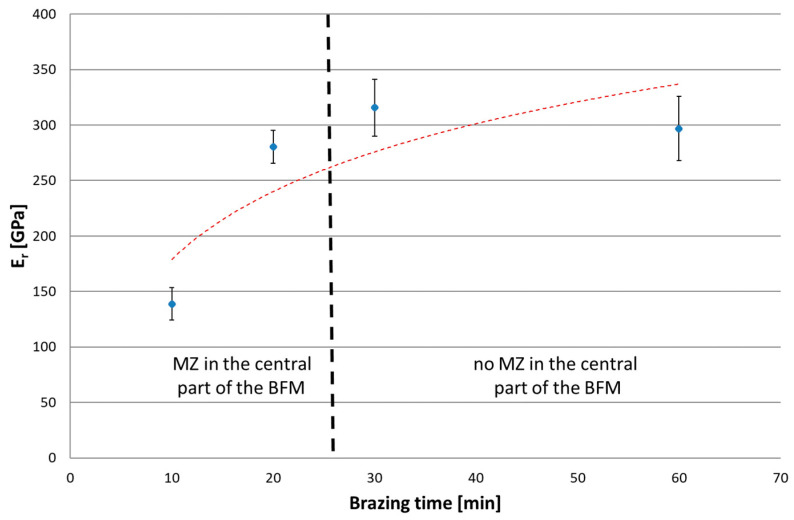
Reduced modulus of the indentation contact for Hastelloy X–Palnicro 36M joints by 1 N load.

**Figure 18:**
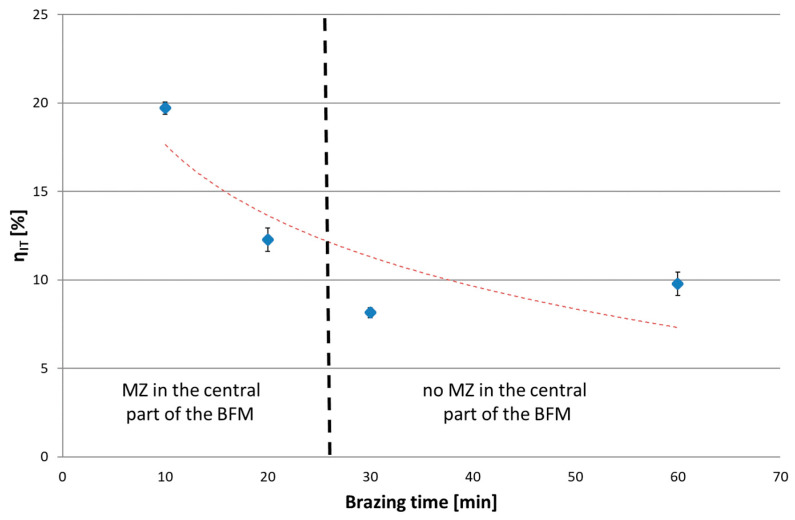
The elastic part of the indentation work for Hastelloy X–Palnicro 36M joints by 1 N load.

**Figure 19:**
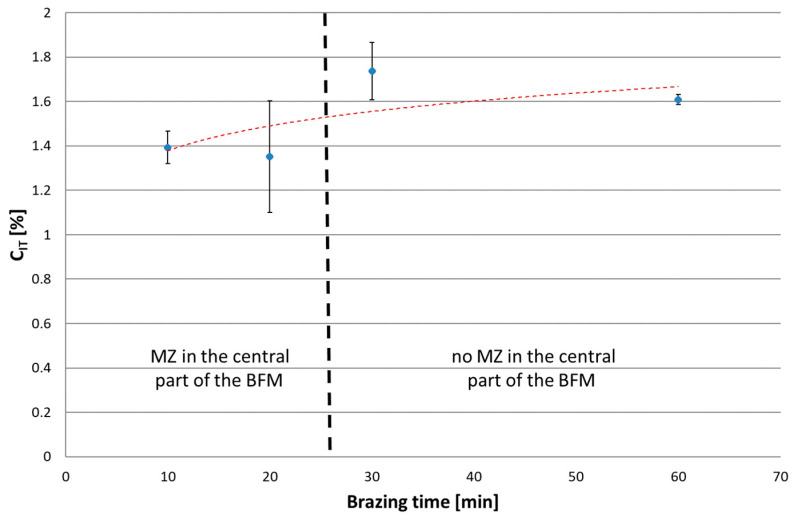
Indentation creep for Hastelloy X–Palnicro 36M joints by 1 N load.

The next series of measurements were taken by increasing the indentation load to 1 N and 2 N/min loading/unloading rate. Increasing the load up to the selected value caused the indenter action on a much wider area of the joint. Tests were conducted for joints with different brazing times (10, 20, 30 and 60 min). Microscopic examination of the size of indentations showed that, for the joints after 10 and 20 min brazing time, the results represent the average properties of SSP and MZ (i.e., matrix, Ni-B, Cr-B and Pd-Ni-Si), while for joints after 30 and 60 min, they represent the homogeneous SSP zone. The results of tests are presented in the following Figure 16, Figure 17, Figure 18 and Figure 19. The ranges of both groups of joints are marked on them: those with a phase-differentiated structure contained MZ and others with a single phase SSP structure.

## 4. Discussion

As could be expected based on the earlier obtained results of wettability and spreadability tests [24], as well as on own previous research [27,52], sound joints of Hastelloy X with the use of Palnicro 36M BFM can be easily obtained. Their structure is complex and, similar to the joints of other Ni superalloys brazed with BNi and other BFMs, and also similar to the joints of the same material obtained by the use of other BFMs without Pd reported in [8,13,17,25,46], it consists of a homogeneous SSP zone, a centrally located MZ with hard precipitates and an intermediate DAZ layer, also with hard precipitates embedded in a more ductile matrix. 

The obtained structure depends on the brazing parameters employed. Depending on the clearance size and brazing time, there are regions free of inclusions for which the joint structure consists of SSP and DAZ only, due to diffusion of boron from liquid BFM to BM that reduces its concentration in the bulk up to, at most, the solubility limit. The total MBC increases from 26.6 μm to 54.4 μm in the adopted range of brazing times. The initial MBC increment rate is about 1 µm/min and decreases as brazing time increases to the value of about 0.4 µm/min.

An analogous relationship to that of the wedge test occurs when changing the gap width for the same brazing conditions (Figure 5), where the share and character of MZ varies from a wide and continuous layer, through intermittent, to its complete disappearance in the interval of 90–45 µm. Comparable behaviour of Hastelloy X joints, however brazed with BNi2, was reported by Malekan et al. [53,54].

The comparison of the linear distributions of the concentration of elements in Figure 4 clearly shows a difference between the joints obtained by various parameters: the sample obtained in a shorter brazing time contains a band of phase precipitations in the middle part, in contrast to the sample after 30 min of brazing. It is made of chromium and nickel borides along with a Pd-Ni-Si phase, which is indicated by the measurements of chemical content using EDS (Table 2) and peaks of elements accumulation at the concentration lines. Direct measurement of the boron content in the MZ region was not possible due to the limitations of the SEM/FESEM-EDX method, despite the optimization of the accelerating voltage to 5 keV, as is suggested in [50]. However, the identification of the Ni-B and Cr-B phases (respectively, the dark gray phase and the fine black phase in Figure 7) was possible on the basis of detecting the presence of this element in the appropriate areas of MZ (Table 2), analysis of the relevant equilibrium phase systems [33,34,35,36,37,38] and macroscopic XRD analysis of this zone (Figure 8). In addition, this is also indicated by the results of the hardness measurements (Figure 12). It should be noted that we are dealing with a five-component BFM system, in which there is a mutual transfer of components with the Hastelloy X substrate during brazing. There is a possibility of formation of a number of compounds, secondary solutions and non-equilibrium structures. For these reasons, the following designations of MZ structural components were applied for the indentation tests: Ni-B, Cr-B, Pd-Ni-Si and “matrix”, without specifying their stoichiometry. The analysis of the concentration curves in Figure 4 points out that, with the extension of the brazing time, the decomposition of the already formed borides occurs with the subsequent diffusion of B to the substrate. Boron compounds Cr_5_B_3_ and Cr-Mo-B and the possible formation of phases based on Cr_3_NiB_6_ were revealed in DAZ in our earlier work [52], which confirm the diffusion of B into the substrate. On the other hand, the presence of Fe in the joints indicates the solubility of the substrate in the liquid BFM in the course of brazing.

In regard to mechanical measurements, the presence of precipitations in DAZ increases the indentation hardness by more than 2 GPa (220 HV) in relation to SSP, and by almost 5 GPa (450 HV) in relation to BM. However, the highest values were recorded for the phases in MZ: Ni-B and Pd-Ni-Si prove 20.5 GPa (1930 HV) and 9.4 GPa (890 HV), respectively. In turn, significant changes are also noticed for the reduced modulus of the indentation contact. The lowest values of 282 ÷ 285 GPa were recorded for BM, Ni-B and Pd-Ni-Si in the MZ. The increase in the E_r_ by about 25 GPa was registered for the BM-DAZ and the matrix in the MZ. The highest values of that modulus above 335 GPa are noted for both DAZ-BFM and SSP zones.

The elastic part of the deformation work ranges from about 7% for BM to about 40% for particular parts of the joint (Figure 14). Ni-B in MZ has the highest η_IT_ value calculated. The lowest values of η_IT_ were obtained for the phases of lower hardness: BM, SSP and the matrix in MZ. The presence of hard phases in DAZ results in an increase in the participation in work of elastic reverse deformation. 

Component parts in aircraft engines often work at elevated temperatures, in areas exposed to vibrations and in the field of tensile stresses. Therefore, their joints, depending on application, must show adequate resistance to fatigue and creep. According to the obtained IIT results (Figure 15), BM is characterized by the highest creep susceptibility at room temperature. The remaining elements of the brazed joint have similar creep resistance, except Ni-B and Pd-Ni-Si in MZ, which are slightly less prone to creep. Therefore, the suggestion arises that brazed Hastelloy X joints using Palnicro 36M are more creep-resistant than the parent material, whether or not there is MZ in their central area. This requires verification, as the behavior of the joint as a whole does not necessarily have to be the superposition of the behavior of its individual components. Moreover, this is a conclusion for the ambient temperature only; it requires authentication for elevated temperatures.

In an IIT test with a load of 30 mN, higher hardness and lower reduced modulus of indentation contact were obtained for Ni-B and Pd-Ni-Si in relation to the SSP. Therefore, in the test with a higher P_max_, the presence of MZ in the BFM increases H_IT_ by 0.8 GPa (75 HV), and decreases E_r_ by more than 150 GPa. In turn, lower creep susceptibility of both mentioned phases decreases the C_IT_ of the whole joints by 0.2%. The relation W_elastic_/W_total_ was also determined for P(h) curves with a load of 1 N, in analogy to the analysis of results for individual phases by 30 mN load. The presence of MZ in joints increases η_IT_. It was observed an excessive scatter of results for 20 min brazing time due to the specific structure of these joints with some intermittent MZ areas in the central part containing basically SSP.

## 5. Conclusions

1. Brazed Hastelloy X joints can be obtained relatively easy with Ni-Cr-based BFM with a high proportion of palladium and additions of melting point depressants (Palnicro 36M). The structure of the joints can be controlled by the brazing time and gap size at a constant brazing temperature. The width range of the joints of a solid solution character without brittle phases (MBC) varies from approx. 20 µm to approx. 50 µm in the brazing times interval from 10 min to 60 min for 995 °C brazing temperature.
2. High Pd content in BFM, despite the fact that it forms an unlimited liquid and solid solution with Ni, does not fundamentally change the formation of the DAZ, SSP and MZ zones known from other systems. However, the appearance of additional hard precipitates with Pd participation (Pd-Ni-Si) was noted.
3. The mechanical properties (indentation and Martens hardness, reduced modulus, elastic part of indentation work and indentation creep) of structural components were tested using the IIT method with a load of 30 mN and the entire joint zones with a 1 N load. The hardest components were Ni-B and Cr-B boride phases, and the Pd-Ni-Si phase in MZ and the boundary of DAZ and BM. MZ reduces the plasticity of the joint to the highest extent.
4. With the increase in brazing time, the hardness of the joints and the elastic part of the indentation work decreased, while the reduced modulus of the indentation contact and indentation creep increased.
5. All structural components of the joints were less susceptible to creep in ambient temperature (indentation creep for SSP, MZ matrix and DAZ in range 7–8%, for Ni-B and Pd-Ni-Si below 6%) than the parent material (indentation creep > 9%).

## Figures and Tables

**Table 1:** Nominal chemical composition and melting temperature range of Hastelloy X [45] and Palnicro-36M [29].

Alloy	Nominal Composition, wt.%	Solidus, °C	Liquidus, °C
Hastelloy X	Ni (balance), 22 Cr, 18 Fe, 9 Mo, 1.5 Co, 0.6 W, 0.1 C, <1 Mn, <1 Si, <0.008 B, <0.5 Nb, <0.5 Al, <0.15 Ti	1260	1355
Palnicro 36M	Ni (balance), 36 Pd, 10.5 Cr, 3 B, 0.5 Si	820	960

**Table 2:** Composition of constituents in MZ and SSP zones in at. % (SEM/FESEM–EDS results).

Zone	Phase	B	Si	Cr	Ni	Pd	Fe	Mo
MZ	Fine black phase (Cr-B compound) *	present	present	present	present	-	-	present
	Dark gray phase (Ni-B compound) *	present	-	-	present	-	-	-
	White phase (Pd-Ni-Si compound)	-	22.4	5.8	10.3	60.9	0.6	-
	Matrix (solid solution)	-	4.1	17.7	43.7	32.3	2.2	-
SSP	Solid solution	-	1.5	15.9	58.0	21.3	3.0	0.3

* Acceleration voltage: 5 kV.

## Data Availability

Not applicable.

## Abbreviations

BM	base material
BFM	brazing filler metal
DAZ	Diffusion-affected zone
SSP	solid solution phase
MZ	multiphase zone
H_IT_	indentation hardness
H_M_	Martens hardness
E_r_	reduced modulus of the indentation contact
η_IT_	elastic part of indentation work
C_IT_	indentation creep
